# RECALLING YOUTH IN VIRTUAL REALITY IN OLDER ADULTS WITH MILD COGNITIVE IMPAIRMENT

**DOI:** 10.1093/geroni/igae098.3703

**Published:** 2024-12-31

**Authors:** DaEun Kim, Hsiao-Wen Liao, Leila Aflatoony, Jeff Wilson

**Affiliations:** Georgia Institute of Technology, Atlanta, Georgia, United States; Georgia Institute of Technology, Atlanta, Georgia, United States; Georgia Institute of Technology, Atlanta, Georgia, United States; Georgia Institute of Technology, Atlanta, Georgia, United States

## Abstract

As people age, their autobiographical memory becomes less vivid and detailed. We developed a virtual reality (VR) environment that recreates a nostalgic lounging area from the 1970s. Aside from examining feasibility, we tested whether cognitively healthy older adults (n = 16; Mage = 66.7) and individuals with mild cognitive impairment (MCI; n = 12; Mage = 75.3) differ in VR experience, memory phenomenology, and memory details, and whether VR experience correlates with memory variables. The virtual lounge comprised standard cues and was customized with personal photos. After the VR session, participants rated VR experience (i.e., presence, time travel, and bringing back memories), described a positive memory from their youth, and rated memory phenomenology (i.e., vividness, reliving, and significance). Memories were content-coded for semantic and episodic details. Regarding feasibility, most participants were able to follow the instructions to navigate the environment, use the controller to turn on a virtual lamp and record player (healthy controls: 100%, 91.7%, 86.7%; MCIs: 75%, 90.9%, 90%), and reported low discomfort in VR. Mann-Whitney’s U tests indicated no group differences in VR experience, memory phenomenology, and semantic details. Healthy controls recalled more episodic details than the MCI group. Overall, sensing presence and experiencing time travel in VR correlated with stronger memory reliving. The extent to which VR brought back memories correlated with greater memory significance. Similar levels of VR experience and memory phenomenology between the two groups, along with the correlations between the two variables, suggest the potential utility of VR for facilitating memory recollection.

